# Age-Related Disparities in the Predictive Performance of the Shock Index for Massive Transfusion in Trauma Patients: A Retrospective Cohort Study

**DOI:** 10.3390/jcm14072416

**Published:** 2025-04-01

**Authors:** Young Mo Cho, Sungwook Park

**Affiliations:** Department of Emergency Medicine, Pusan National University Hospital, 179, Gudeok-ro, Seo-Gu, Busan 49241, Republic of Korea; specialkey7@naver.com

**Keywords:** shock, hemorrhage, aged, blood transfusion, risk assessment

## Abstract

**Background:** In trauma, the shock index (SI) is commonly used to assess the presence of significant blood loss. Prior studies have shown that the SI has a fair predictive ability for clinical outcomes such as massive transfusion (MT) or mortality in adult trauma patients. We hypothesized that the relatively lower predictive power of the SI in older adult patients compared to that of younger adult patients results in the overall fair predictive ability of the SI for clinical outcomes in adult trauma patients. **Methods:** This retrospective observational study analyzed adult trauma patients who presented to a single regional trauma center between 2019 and 2023, categorizing them into younger (18–64 years) and older (≥65 years) cohorts. The association between SI and MT was evaluated using simple logistic regression, while the modifying effect of age on this association was evaluated through an interaction model. The predictive performance was compared between the groups using the area under the receiver operating characteristic curve (AUC). Age-stratified AUC trends were visualized using cubic spline analysis. **Results:** A total of 2404 trauma patients met the inclusion criteria, including 1531 younger adults and 873 older adults. The SI was identified as an independent predictor of MT, with a stronger association in younger adults. The AUC for predicting MT was significantly higher in younger adults compared to older adults (0.801 vs. 0.666; *p* < 0.001), with optimal SI cut-off values of 1.18 and 0.88, respectively. Age-stratified analysis showed the highest AUC in the 41–50 age group (AUC 0.880; 95% CI, 0.836–0.916) and the lowest in the 71–80 age group (AUC 0.624; 95% CI, 0.573–0.674). **Conclusions:** The predictive performance of the SI for MT was influenced by age, demonstrating a lower predictive ability in older adult patients compared to younger adults.

## 1. Introduction

Hemorrhagic shock is a major cause of preventable death among injured patients, accounting for approximately 35% to 40% of all trauma-related mortality, second only to traumatic brain injury [1,2]. Early death in trauma is often due to exsanguination, which is responsible for 44.7% of fatalities occurring in the prehospital setting or emergency department (ED) [2], underscoring the critical need for tools that can accurately identify patients requiring early hemodynamic stabilization interventions, such as massive transfusion (MT) or angiographic embolization.

The shock index (SI), defined as heart rate (HR) divided by systolic blood pressure (SBP), is a simple and effective tool for assessing blood loss. An abnormal SI can indicate underlying shock or critical illness [3,4,5]. In trauma patients, previous studies have shown that the SI can predict MT requirements or higher mortality [6,7,8]. However, one systemic review showed that its overall effectiveness in predicting serious injury in adult trauma patients remains fair, with the pooled area under the receiver operating characteristic curve (AUC) values ranging from 0.71 to 0.72 [9], indicating that it may lack sufficient accuracy to be used as a standalone tool in clinical practice.

Age-related changes, including reduced physiologic reserve, increase the prevalence of chronic diseases, while the use of medications such as antihypertensive or antiarrhythmic agents may impair compensatory responses to hemorrhage. These factors likely diminish the predictive power of SI with increasing age, reducing its effectiveness in older adults. A prior study demonstrated a low predictive performance of the SI for mortality in elderly trauma patients, supporting this inference [10,11]. However, direct comparisons of the predictive capabilities of SI between older and younger adult trauma patients remain scarce. Therefore, this study aimed to compare the predictive performance of the SI for MT between older and younger adult groups. We hypothesized that SI would demonstrate less accurate predictive performance in older adult patients compared to younger adult patients.

## 2. Materials and Methods

### 2.1. Study Design

This was a retrospective observational study conducted at a single trauma center in a 1400-bed tertiary care university-affiliated hospital in Busan, Korea. As a Level I regional trauma center, the facility mainly provides specialized care for patients in Busan City and Gyeongnam Province. Study approval was obtained from the hospital’s institutional review board (IRB) (approval number: 2411-013-145); informed consent was waived because this study was retrospective in nature.

### 2.2. Patients

We included all consecutive adult patients (aged ≥18 years) who presented to the trauma center between 1 January 2019 and 31 December 2023. We excluded patients under 18 years of age, patients transferred from other hospitals, patients who experienced cardiac arrest before arrival at the trauma center, and patients whose admission SBP was too low to obtain a measurement, preventing the calculation of the SI. The included patients were categorized into two groups—older adults (age ≥ 65 years) and younger adults (age < 65 years).

### 2.3. Data Collection

When injured patients arrive at emergency medical facilities nationwide, each facility compiles a trauma registry and sends it to the National Emergency Medical Center. These aggregated data are from the Korean Trauma Data Bank (KTDB). Each patient’s trauma registry is a disease-specific data collection that includes uniform data elements detailing the injury event, prehospital information, demographics, diagnosis, and outcomes.

From the trauma registry recorded at our trauma center for submission to the KTDB, we collected data on age, sex, mechanism of injury, time from injury to trauma center arrival, the time between emergency medical service (EMS) departure from the scene and trauma center arrival, prehospital fluid administration, initial vital signs at ED admission (HR, SBP, and diastolic pressure), injury severity score (ISS), body regions with abbreviated injury scale (AIS) ≥ 3, total Glasgow Coma Scale (GCS) score, the number of units of transfused packed red blood cells in the first 24 h of admission to the trauma center, intensive care unit admission, and in-hospital mortality.

Additionally, from the electronic medical records, we obtained laboratory values, including hemoglobin (Hb), platelet (PLT), prothrombin time international normalized ratio (PT INR), and ethanol, as well as arterial blood gas analysis (ABGA) findings such as pH, base excess (BE), and lactate. Arterial and venous blood samples for these laboratory values were collected immediately upon the patient’s arrival at the trauma center.

### 2.4. Outcome

The outcome of this study was MT, defined as the transfusion of ≥10 units of PRBCs within 24 h of arrival at the trauma center.

### 2.5. Statistical Analysis

Continuous variables were presented as medians and interquartile ranges (IQRs), and normality was assessed using the Kolmogorov–Smirnov test. Categorical variables were summarized as counts and proportions. Group comparisons were performed using the Mann–Whitney U test for continuous variables and the Chi-squared test or Fisher’s exact test for categorical variables.

Logistic regression analyses were performed to evaluate the association between the SI and MT using both a simple model and an interaction model. The simple model included SI, age group (age ≥ 65 or age < 65), and other covariates that showed significant differences between the non-MT and MT groups in the univariate analysis. These covariates included injury mechanisms, GCS score, hemoglobin, platelet count, PT INR, lactic acid (≥4 or <4), time from injury to trauma center arrival, fluid administration (yes/no), and body regions with AIS ≥ 3. The interaction model included an additional interaction term (SI × age group) to assess whether age modified the association between SI and MT. Adjusted odds ratios (AORs) with 95% confidence intervals (CIs) were calculated for both models.

Receiver operating characteristic (ROC) curve analysis was performed to evaluate the predictive performance of the SI for MT, with the AUC quantifying its overall predictive ability. Optimal cut-off values were determined using the Youden index within each age group, based on the entire study cohort in each respective age group, without internal validation, given the large sample size. To investigate age-related differences in the SI’s predictive performance, the AUC values between younger adults and older adults were compared using DeLong’s test. In addition, to explore trends in the relationship between age and the predictive performance of the SI, cubic spline interpolation was conducted by stratifying patients into 10-year age increments.

A two-sided *p*-value of <0.05 was considered statistically significant. All statistical analyses were performed using IBM SPSS Statistics for Windows, version 21.0 (IBM Corp., Armonk, NY, USA) and MedCalc for Windows, version 23.0.5 (MedCalc Software Ltd., Ostend, Belgium).

## 3. Results

### 3.1. Comparison of Patient Characteristics and Outcomes

A total of 2404 patients (median age [IQR]: 59.0 [43.0–69.0] years) were included. Of these, 873 (36.2%) were younger adult patients and 1531 (63.8%) were older adult patients. The baseline characteristics for these age groups are summarized in Table 1.

**Figure 1:**
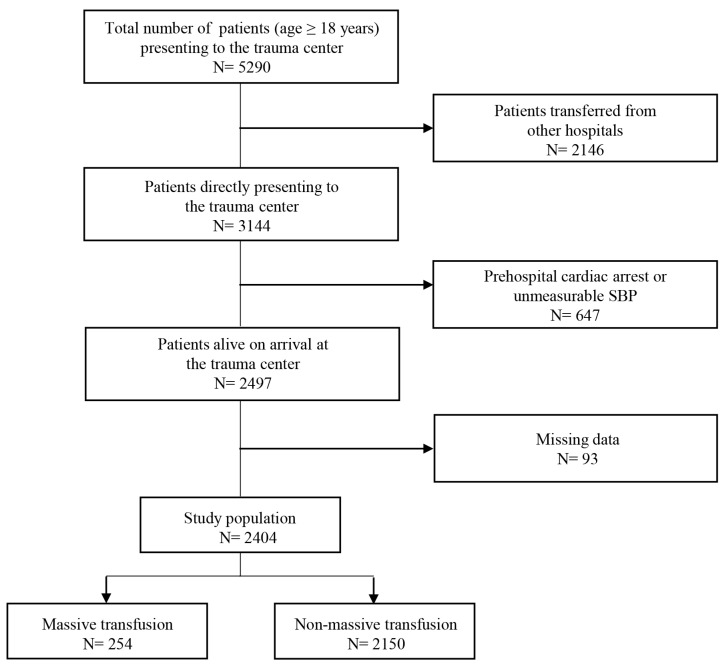
Study flow diagram of adult trauma patients presenting to our regional trauma center. SBP: systolic blood pressure.

Based on the number of transfused PRBC units, 254 patients (10.6%) were classified into the MT group and 2150 patients (89.4%) into the non-MT group, as depicted in Figure 1.

The median age was significantly higher in the MT group (63.0 years [IQR: 47.8–73.0]) compared to the non-MT group (58.0 years [IQR: 43.0–69.0]) (*p* < 0.001). The proportion of males was slightly lower in the MT group (71.7%) compared to the non-MT group (75.5%) (*p* = 0.192). Traffic accidents were the most common mechanism of injury in both groups, accounting for 55.9% of injuries in the MT group and 45.2% in the non-MT group. Falls from height were more frequent in the non-MT group, while cutting or piercing injuries were more common in the MT group.

The time from injury to trauma center arrival was significantly shorter in the MT group (*p* = 0.008), and EMS fluid administration was significantly higher (*p* < 0.001). The MT group had significantly lower admission systolic blood pressure (*p* < 0.001) and hemoglobin levels (*p* < 0.001) but higher lactate levels (*p* < 0.001). Severe injuries (AIS ≥ 3) were more prevalent in the MT group, particularly in the thorax, abdomen/pelvic contents, and lower extremities. The median ISS was significantly higher in the MT group, and in-hospital mortality was substantially higher than in the non-MT group (*p* < 0.001) (Table 2).

### 3.2. Logistic Regression Analyses for the Association Between SI and MT

In the simple model, SI was identified as a strong independent predictor of MT (aOR 2.49; 95% CI 1.77–3.51; *p* < 0.001). In the interaction model, the interaction term indicated that aOR = 0.52, 95% CI = 0.28–0.96, and *p* = 0.007, indicating that the association between SI and MT was significantly modified by age. Specifically, the association was stronger in younger adults (aOR 3.91; 95% CI 2.62–5.86) compared to older adults (aOR 2.02; 95% CI 1.09–3.76).

### 3.3. ROC Analysis of SI for Predicting MT

**Figure 2:**
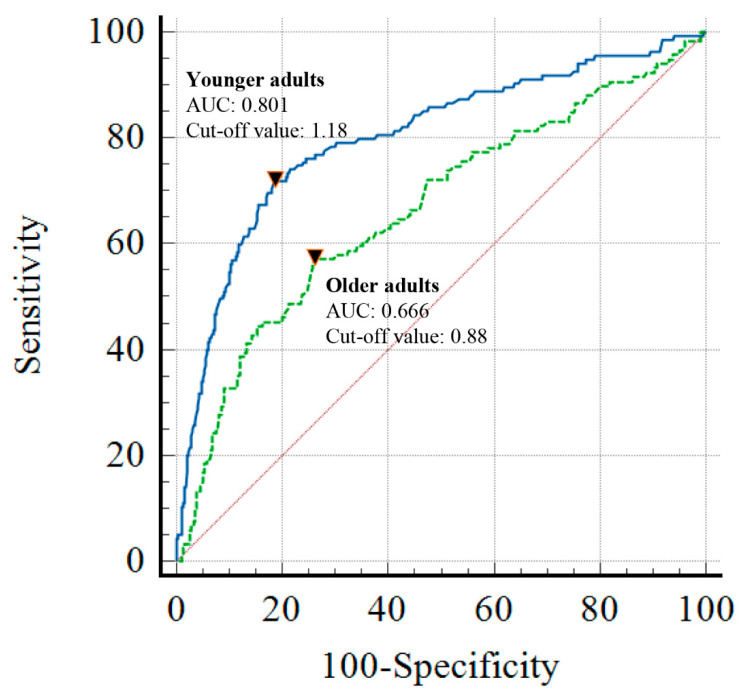
ROC curves for massive transfusion in the younger and older adult groups. The inverted triangles indicate the Youden index. Abbreviations—ROC: receiver operating characteristics; AUC: area under the curve.

In the ROC curve analysis, the AUC of the SI for predicting MT in all study patients was 0.718, with a cutoff value of 1.06 (sensitivity: 61.4%; specificity: 78.6%). When the analysis was stratified into two groups (age ≥ 65 and <65 years), the AUC of the SI was significantly higher in the younger adult group compared to the older adult group (0.801 vs. 0.666; *p* < 0.001). The optimal SI cut-off values also differed between the two groups, i.e., 1.18 (sensitivity: 71.9%; specificity: 81.5%) for younger adults and 0.88 (sensitivity: 57.1%; specificity: 74.0%) for older adults. These results are depicted in Figure 2. 

**Figure 3:**
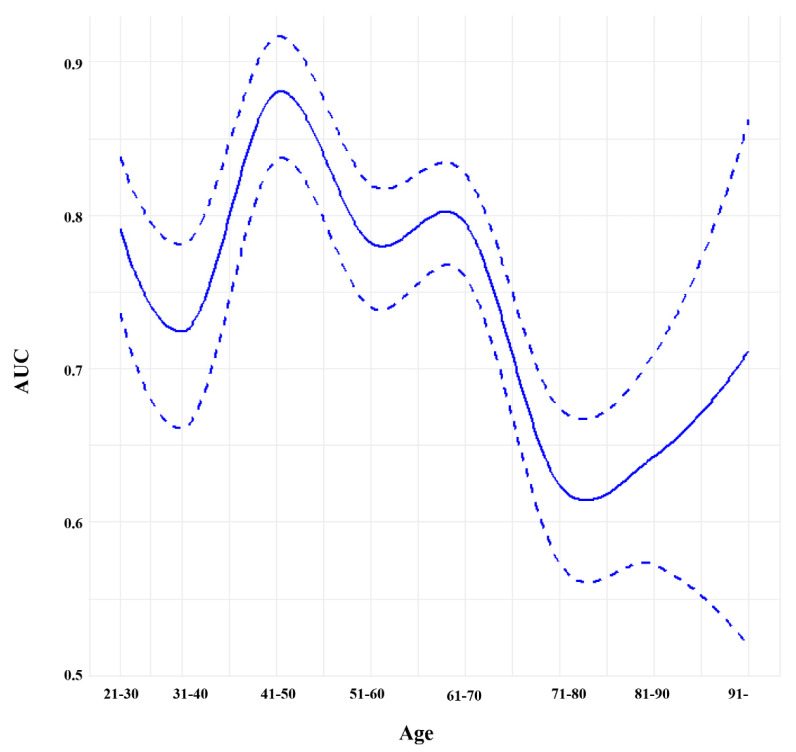
Predictive performance of the shock index stratified by age. The relationship between age and the predictive performance of the SI was stratified by 10-year increments. The solid line represents the AUC, while the dashed line indicates the 95% confidence interval. Abbreviations—SI: shock index; AUC: area under the curve.

When further stratified according to 10-year age increments, it was revealed that the relationship between age and the predictive performance of the SI demonstrated a non-linear trend, as visualized in Figure 3. The highest predictive performance was observed in the 41–50 age group (AUC 0.880; 95% CI 0.836–0.916), while the lowest was seen in the 71–80 age group (AUC 0.624; 95% CI 0.573–0.674).

## 4. Discussion

In this study, we found that while the SI was a reliable predictor of MT overall, its accuracy was lower in older adults compared to younger adults and declined with advancing age. In addition, the optimal cut-off values of SI for predicting MT differed between groups. These findings suggest that the application of the SI in clinical practice should differ between older and younger adults, with consideration being given to age-specific thresholds.

This study found that the accuracy of the SI for predicting MT in all study patients was fair (AUC = 0.718). This result is consistent with a systematic review that reported pooled AUC values of 0.72 (95% CI 0.66–0.77) for out-of-hospital SI based on seven studies and 0.71 (95% CI, 0.66–0.76) for ED SI based on eleven studies [9]. However, a more recent systematic review of 15 studies assessing the predictive ability of SI for MT reported an overall sensitivity of 0.68 (95% CI 0.57–0.76) and an overall AUC of 0.85 (95% CI 0.81–0.88) [12], which exceeds our results. Interestingly, for secondary outcomes of mortality prediction, the predictive capacity of the SI was notably lower, with an AUC of 0.553 [12]. The variability in the predictive ability of the SI may stem from differences in our study populations (e.g., inclusion of geriatric patients or exclusion of patients with traumatic brain injuries) and outcomes, such as MT or mortality. Moreover, the context in which the SI is applied—whether prehospital or hospital—also appears to influence its performance. Although the overall predictive ability of the SI remains inconsistent across studies, one common finding from previous and current research is that the SI has relatively low sensitivity, increasing the potential risk of under-triage.

The present study found that when stratified by age group, the aOR in younger adults was higher than in older adults. The ROC analysis further underscored this disparity, demonstrating significantly higher predictive performance in younger adults (AUC = 0.801) compared to older adults (AUC = 0.666). More specifically, the AUC declined progressively with advancing age, with the lowest performance noted in the 71–80 age group. These findings suggest a reduced predictive utility of SI in older adults, potentially limiting its reliability as a predictive tool in this population. Similar age-related patterns have been reported in previous studies. Shibahashi et al. [10] using a large dataset from the Japan Trauma Data Bank, identified a progressive age-related decline in SI’s predictive performance for MT, with AUC values decreasing from 0.788 in patients aged 20–24 years to 0.660 in those aged 80–84 years [10]. Similarly, Zarzaur et al. [11] reported lower AUC values for SI in older adults (≥55 years) compared to younger adults (<55 years) for predicting 48-h mortality (0.789 vs. 0.856). Their study further suggested that multiplying SI by patient age in those >55 years could enhance its predictive utility for early mortality and blood transfusion needs [11]. This age-related disparity in SI’s predictive ability may be partly explained by physiological and pharmacological factors unique to older adults. Chronic conditions such as hypertension and coronary artery disease, along with medications like beta-blockers and calcium channel blockers, may dampen the dynamic relationship between HR and SBP, thereby reducing SI’s utility [13]. Furthermore, older adults typically exhibit a diminished HR response to physiological stressors, which could further limit the effectiveness of SI in this population [14].

However, contradictory findings exist. DeMuro et al. [15] reported a similar sensitivity of SI across age groups for identifying patients requiring hemostatic intervention but observed greater specificity in older patients (≥65 years), suggesting that SI may perform better in older populations [15]. Likewise, Rafieezadeh et al. [16] analyzed data from 244,943 patients and divided patients into three age groups (25–44, 45–64, and ≥65 years). This study showed that for every 0.1 increase in SI, the odds of mortality, blood transfusion, and major surgical intervention increased across all age groups, with the strongest effect observed in the oldest cohort [16]. Moreover, significantly higher AUC values were found for SI in older adults (≥65 years) compared to younger groups for predicting major surgical interventions or blood transfusion needs [16]. Pandit et al. [17] in an analysis of 217,190 geriatric trauma patients from the National Trauma Data Bank, demonstrated that patients with an SI ≥ 1 were more likely to require blood transfusions and exploratory laparotomies. Furthermore, an SI ≥ 1 was identified as a strong independent predictor of mortality with high accuracy [17]. These studies differed in terms of the selection of patient criteria, outcome measures, and statistical methodologies. For instance, the study of Zarzaur et al. [11] included only blunt trauma patients and excluded those with head or spinal cord injury, unlike other studies that included all trauma patients. In the studies of Shibahahi et al. [10] and Zarzaur et al. [11] the primary outcome was mortality, whereas in other studies, the primary outcomes were the need for hemostatic intervention and blood transfusion. These methodological differences, in addition to the limited number of studies specifically investigating the impact of age on the predictive performance of the SI, may contribute to the contradictory findings across the studies. Moreover, although SI has been recommended to assess transfusion requirements or to be used as one of the components to predict severe injury, current guidelines do not account for age [18,19]. Given that studies have reported that SI may not be reliable in geriatric patients [20,21], further research investigating the effect of age on the predictive performance of SI is warranted.

The strength of our study is that we included a large cohort and employed diverse statistical methods to examine how age moderates the predictive utility of SI. Although several studies have reported age-related declines in SI’s predictive performance, most of these studies relied on simple measures, such as odds ratios or AUC values, for either overall adult or geriatric populations. These measures can demonstrate simple associations between age and the performance of SI, but they are insufficient to explain how the performance of SI changes with increasing age.

This study has several limitations that should be considered when interpreting the findings. First, selection bias may exist for two reasons. Our study was conducted at a single regional trauma center (Level I) that primarily treats patients meeting the “red criteria” defined in the National Guidelines for the Field Triage of Injured Patients [19], representing a cohort with more severe injuries compared to those treated at lower-level trauma centers or non-trauma hospitals. In addition, patients who experienced prehospital cardiac arrest or had an unmeasurable SBP were excluded from the analysis. Although this exclusion ensured data consistency, it may have introduced bias by omitting the most critically ill patients, potentially leading to an overrepresentation of patients with more stable hemodynamics. Second, this study stratified patients into two age groups in accordance with definitions widely used in previous trauma studies. However, significant physiological heterogeneity may exist within the older adult population, and a simple two-group stratification may not fully capture these differences. To address this limitation, we performed additional age-stratified analysis using 10-year age increments and cubic spline interpolation. Nevertheless, future studies using more detailed multi-group stratification may provide additional insights. Third, we could not collect data on potential confounders such as pre-existing comorbidities, frailty, or medication use; thus, we could not include them in the statistical analysis. This was because many patients were in critical condition at presentation or were unable to provide accurate information regarding their medical history and current medications. Consequently, there were substantial missing data for certain variables, and these factors may have affected the predictive performance of SI in older adults. Fourth, we focused solely on the impact of age on the predictive performance of SI for MT. Although various trauma scores, such as the Assessment of Blood Consumption score, Trauma-Associated Severe Hemorrhage Score, or Massive Transfusion Score, can demonstrate better predictive performance than the SI by incorporating laboratory or imaging findings along with vital signs, these additional requirements limit their feasibility for use in prehospital or early emergency department triage. Therefore, we determined that identifying factors affecting the predictive performance of the widely used SI in clinical practice was more appropriate than directly comparing it with other complex scores that are less practical for early triage. Fifth, during our study period, no standardized protocols based solely on SI thresholds were applied; therefore, we could not assess how SI-guided interventions impact patient outcomes such as mortality, ICU length of stay, or complications.

## 5. Conclusions

In this study, the SI was an independent predictor of MT in trauma patients. The predictive ability of the SI decreased with advancing age, with older adults demonstrating a lower predictive performance compared to that of younger adults. Furthermore, differences in optimal SI cut-off values between younger and older adults suggest the need for age-specific thresholds in clinical practice.

## Figures and Tables

**Table 1:** Baseline characteristics of the younger and older adult groups.

	Younger Adult (n = 1531)	Older Adult (n = 873)	*p*-Value
Age, years	49.0 (33.0–58.0)	73.0 (69.0–80.0)	<0.001
Males, n (%)	1203 (78.6)	603 (69.1)	<0.001
Mechanism of injury, n (%)			<0.001
Traffic accident	710 (46.4)	403 (46.2)	
Fall from height	427 (27.9)	284 (32.5)	
Ground-level fall	36 (2.4)	57 (6.5)	
Struck by object	100 (6.5)	32 (3.7)	
Cutting or piercing	238 (15.5)	78 (8.9)	
Others	20 (1.3)	19 (2.2)	
Time from injury to TC arrival, (min)	47.0 (35.0–64.0)	46.5 (35.0–66.0)	0.839
Time from arrival of EMS on scene to TC arrival, (min)	9.0 (6.0–13.0)	9.0 (6.0–13.0)	0.692
EMS fluid administration, n (%)	206 (13.5)	83 (9.5)	0.058
Admission vital signs			
SBP (mmHg)	110.0 (90.0–130.0)	120.0 (90.0–150.0)	<0.001
DBP (mmHg)	70.0 (60.0–80.0)	80.0 (60.0–100.0)	<0.001
HR (beats/min)	92.0 (78.0–11.0)	87.0 (74.0–101.0)	<0.001
Shock index	0.85 (0.66–1.14)	0.70 (0.54–0.95)	<0.001
Shock index > 1, n (%)	498 (32.5)	194 (22.2)	<0.001
Age shock index	37.3 (26.8–52.6)	51.7 (40.5–73.1)	<0.001
Total Glasgow Coma Scale score	15.0 (11.0–15.0)	14.0 (8.0–15.0)	<0.001
Admission laboratory findings			
Hemoglobin (g/dL)	13.6 (12.3–14.8)	12.4 (11.0–13.7)	<0.001
Platelet (×10^3^/μL)	256.0 (214.0–300.0)	207.0 (168.0–245.3)	<0.001
PT INR	1.05 (1.00–1.12)	1.07 (1.01–1.16)	<0.001
pH	7.39 (7.34–7.43)	7.41 (7.36–7.45)	<0.001
Lactate (mmol/L)			
>4.0, n (%)	488 (32.7)	220 (25.8)	0.069
AIS ≥ 3 injuries to body regions			
Head	521 (34.0)	409 (46.8)	<0.001
Face	12 (0.8)	5 (0.6)	0.553
Neck	12 (0.8)	6 (0.7)	0.792
Thorax	548 (35.8)	315 (36.1)	0.887
Abdomen/Pelvic contents	234 (15.3)	98 (11.2)	0.006
Spine	126 (8.2)	81 (9.3)	0.378
Upper extremities	59 (3.9)	21 (2.4)	0.057
Lower extremities	324 (21.2)	149 (17.1)	0.015
Outcomes of trauma			
ISS	17.0 (10.0–26.0)	20.0 (13.0–27.0)	<0.001
ISS ≥ 16, n (%)	935 (61.1)	602 (69.0)	<0.001
1 h PRBC, units	0 (0–2.0)	1.0 (0–3.0)	<0.001
1 h PRBC ≥ 3 units, n (%)	251 (16.4)	196 (22.5)	<0.001
24 h PRBC, units	1.0 (0–4.0)	2.0 (0–6.0)	<0.001
24 h PRBC ≥ 10 units, n (%)	135 (8.8)	119 (13.6)	<0.001
In-hospital mortality, n (%)	118 (7.7)	215 (24.6)	<0.001

EMS: emergency medical service; TC: trauma center; SBP: systolic blood pressure; DBP: diastolic blood pressure; HR: heart rate; PT INR: prothrombin time international normalized ratio; ISS: injury severity score.

**Table 2:** Baseline characteristics of massive transfusion and non-massive transfusion groups.

	MT (n = 254)	Non-MT (n = 2150)	*p*-Value
Age, years	63.0 (47.8–73.0)	58.0 (43.0–69.0)	<0.001
Males, n (%)	182 (71.7)	1624 (75.5)	0.192
Mechanism of injury, n (%)			0.002
Traffic accident	142 (55.9)	971 (45.2)	
Fall from height	66 (26.0)	645 (30.0)	
Ground-level fall	3 (1.2)	90 (4.2)	
Struck by object	19 (7.5)	113 (5.3)	
Cutting or piercing	21 (8.3)	295 (13.7)	
Others	3 (1.2)	36 (1.7)	
Time from injury to TC arrival, (min)	43.0 (33.0–57.0)	46.0 (34.0–64.0)	0.008
Time from arrival of EMS on scene to TC arrival, (min)	8.0 (6.0–12.0)	9.0 (6.0–13.0)	0.098
EMS fluid administration, n (%)	54 (21.3)	235 (10.9)	<0.001
Admission vital signs			
SBP (mmHg)	80.0 (60.0–110.0)	110.0 (90.0–140.0)	<0.001
HR (beats/min)	104.0 (84.0–122.3)	89.0 (76.0–105.0)	<0.001
Shock index	1.3 (0.8–1.7)	0.8 (0.6–1.0)	<0.001
Shock index category, n (%)			<0.001
<0.7	45 (17.7)	842 (39.2)	
0.7–1.0	43 (16.9)	782 (36.4)	
>1.0	166 (65.4)	526 (24.5)	
Total Glasgow Coma Scale score	10.0 (5.0–15.0)	15.0 (11.0–15.0)	<0.001
Admission laboratory findings			
Hemoglobin (g/dL)	11.4 (10.2–13.0)	13.4 (12.0–14.6)	<0.001
Platelet (×10^3^/μL)	206.5 (157.8–251.0)	241.0 (197.0–284.0)	<0.001
PT INR	1.21 (1.11–1.37)	1.04 (0.99–1.11)	<0.001
pH	7.36 (7.28–7.42)	7.40 (7.36–7.44)	<0.001
Lactate (mmol/L)			
>4.0, n (%)	139 (54.7)	569 (27.1)	<0.001
AIS ≥ 3 injuries to body regions			
Head	127 (50.0)	803 (37.3)	<0.001
Face	6 (2.4)	11 (0.5)	0.006
Neck	3 (1.2)	15 (0.7)	0.428
Thorax	139 (54.7)	724 (33.7)	<0.001
Abdomen/Pelvic contents	84 (33.1)	248 (11.5)	<0.001
Spine	22 (8.7)	185 (8.6)	1.000
Upper extremities	10 (3.9)	70 (3.3)	0.577
Lower extremities	92 (36.2)	381 (17.7)	<0.001
Outcomes of trauma			
ISS	29.0 (22.0–36.0)	17.0 (10.0–25.0)	<0.001
ISS ≥ 16, n (%)	236 (92.9)	1301 (60.5)	<0.001
In-hospital mortality, n (%)	113 (44.5)	220 (10.2)	<0.001

EMS: emergency medical service; TC: trauma center; SBP: systolic blood pressure; HR: heart rate; PT INR: prothrombin time international normalized ratio; ISS: injury severity score.

## Data Availability

The datasets analyzed during the current study are available from the corresponding author upon reasonable request.
